# Artificial intelligence in ischemic stroke images: current applications and future directions

**DOI:** 10.3389/fneur.2024.1418060

**Published:** 2024-07-10

**Authors:** Ying Liu, Zhongjian Wen, Yiren Wang, Yuxin Zhong, Jianxiong Wang, Yiheng Hu, Ping Zhou, Shengmin Guo

**Affiliations:** ^1^School of Nursing, Southwest Medical University, Luzhou, China; ^2^Department of Oncology, The Affiliated Hospital of Southwest Medical University, Luzhou, China; ^3^Wound Healing Basic Research and Clinical Applications Key Laboratory of Luzhou, Southwest Medical University, Luzhou, China; ^4^School of Nursing, Guizhou Medical University, Guiyang, China; ^5^Department of Rehabilitation, The Affiliated Hospital of Southwest Medical University, Luzhou, China; ^6^Department of Medical Imaging, Southwest Medical University, Luzhou, China; ^7^Department of Radiology, The Affiliated Hospital of Southwest Medical University, Luzhou, China; ^8^Nursing Department, The Affiliated Hospital of Southwest Medical University, Luzhou, China

**Keywords:** ischemic stroke, medical imaging, deep learning, machine learning, artificial intelligence, prediction model

## Abstract

This paper reviews the current research progress in the application of Artificial Intelligence (AI) based on ischemic stroke imaging, analyzes the main challenges, and explores future research directions. This study emphasizes the application of AI in areas such as automatic segmentation of infarct areas, detection of large vessel occlusion, prediction of stroke outcomes, assessment of hemorrhagic transformation risk, forecasting of recurrent ischemic stroke risk, and automatic grading of collateral circulation. The research indicates that Machine Learning (ML) and Deep Learning (DL) technologies have tremendous potential for improving diagnostic accuracy, accelerating disease identification, and predicting disease progression and treatment responses. However, the clinical application of these technologies still faces challenges such as limitations in data volume, model interpretability, and the need for real-time monitoring and updating. Additionally, this paper discusses the prospects of applying large language models, such as the transformer architecture, in ischemic stroke imaging analysis, emphasizing the importance of establishing large public databases and the need for future research to focus on the interpretability of algorithms and the comprehensiveness of clinical decision support. Overall, AI has significant application value in the management of ischemic stroke; however, existing technological and practical challenges must be overcome to achieve its widespread application in clinical practice.

## 1 Introduction

Ischemic stroke is a prevalent cerebrovascular disease characterized by cerebral ischemia and hypoxia due to an obstruction of blood flow in the brain. It is associated with high rates of disability and recurrence. Globally, stroke is the second leading cause of death and poses a significant threat to human life and health (1). According to the Global Burden of Disease study, the incidence of ischemic stroke worldwide increases every year (2). Rapid and accurate diagnosis, as well as treatment plan selection by clinicians, are crucial for patients with ischemic stroke. Medical imaging is the gold standard for diagnosing ischemic stroke and also aids physicians in choosing treatment plans.

By analyzing the hypodense regions on computed tomography (CT) images of patients with a first episode of stroke, physicians can identify intracerebral hemorrhage and assess for signs of ischemia. CT angiography (CTA) is a contrast-enhanced technique specifically for detecting and evaluating large vessel occlusions (LVO) in the brain and visualizing the status of collateral vessels. CTA involves injecting a contrast and rapidly scanning the brain to capture the dynamic process of the contrast agent passing through the blood vessels, generating time-density curves. These curves record the changes in density over time for each voxel (three-dimensional pixel), thereby allowing the calculation of several key hemodynamic parameters, such as cerebral blood volume (CBV), cerebral blood flow (CBF), and time to peak (TTP). These parameters are crucial for distinguishing between the ischemic penumbra and the necrotic core (3). Magnetic Resonance Imaging (MRI), which mainly employs Diffusion-Weighted Imaging (DWI) and T2-Weighted Fluid-Attenuated Inversion Recovery (FLAIR) sequences, can also help physicians determine the presence of stroke and assess the extent of cerebral infarction. However, it must be combined with clinical manifestations and other examination results to determine the onset and type of stroke more accurately (4). Overall, an objective and accurate evaluation of patients with ischemic stroke poses a significant challenge in current clinical practice. Addressing this challenge is of great importance for the early warning, diagnosis, and treatment of patients at high risk of ischemic stroke.

The rapid development of medical imaging technology has generated a vast amount of highly valuable data with great potential for clinical applications. Consequently, artificial intelligence (AI) technologies, particularly machine learning (ML) and large language models (LLMs), have attracted widespread attention. Their powerful image analysis and information processing capabilities have significant application in various aspects of stroke management, including early diagnosis, prognosis prediction, and automatic segmentation and identification of lesions (5).

The major types of ML are supervised and unsupervised. Supervised learning is currently the most widely used type at the intersection of AI and stroke research. Common supervised learning algorithms include linear regression, logistic regression (LR), random forest (RF), support vector machines (SVM), decision trees, and neural networks. These algorithms train models using known input and output data to predict and classify new data. Traditional ML methods, such as SVM and decision trees, rely on feature engineering, which entails manual extraction, selection, and data-cleaning processes. However, these methods still face challenges in optimizing image features and addressing multimodal image interference (6). In contrast, deep learning (DL) has brought about revolutionary changes in medical image analysis. DL mimics the structure and function of neural networks in the human brain and automatically learns and extracts data features through multilayer neural networks, thereby effectively solving complex problems (7). Compared to classical ML algorithms, DL has more parameters and thus possesses stronger feature representation capabilities. DL has developed multiple technical frameworks based on different data characteristics, among which convolutional neural networks (CNN) are the most widely used. Owing to its multilayer structure, DL has significant advantages in feature representation, generalization, and handling of non-linear problems. DL can automatically learn and extract complex patterns from large datasets, making it particularly suitable for ischemic stroke imaging tasks, such as lesion detection and segmentation, collateral circulation scoring, and identifying the status of LVOs. The integration of DL in stroke management not only enhances diagnostic accuracy, but also aids in the development of personalized treatment plans, ultimately improving patient outcomes.

LLMs constitute an important branch of AI research and their powerful natural language understanding and processing capabilities have attracted widespread attention in the medical field. The Transformer model, built on the self-attention mechanism, is the foundation of LLM research and consists of encoder and decoder structures. This model can effectively identify and process the complex relationships between elements in sequential data, and it performs exceptionally well when handling longer natural language data sequences. Compared with traditional supervised deep learning models, transformer models reduce the need for large amounts of manual annotation while also possessing greater scalability. However, Transformer models often contain numerous parameters and require large-scale datasets to achieve optimal performance. In contrast, CNNs can capture local features through their convolutional layers and maintain their performance on smaller datasets through a parameter-sharing mechanism. Therefore, combining the characteristics of CNNs and Transformer models for specific application scenarios may be a more efficient strategy.

**Figure 1:**
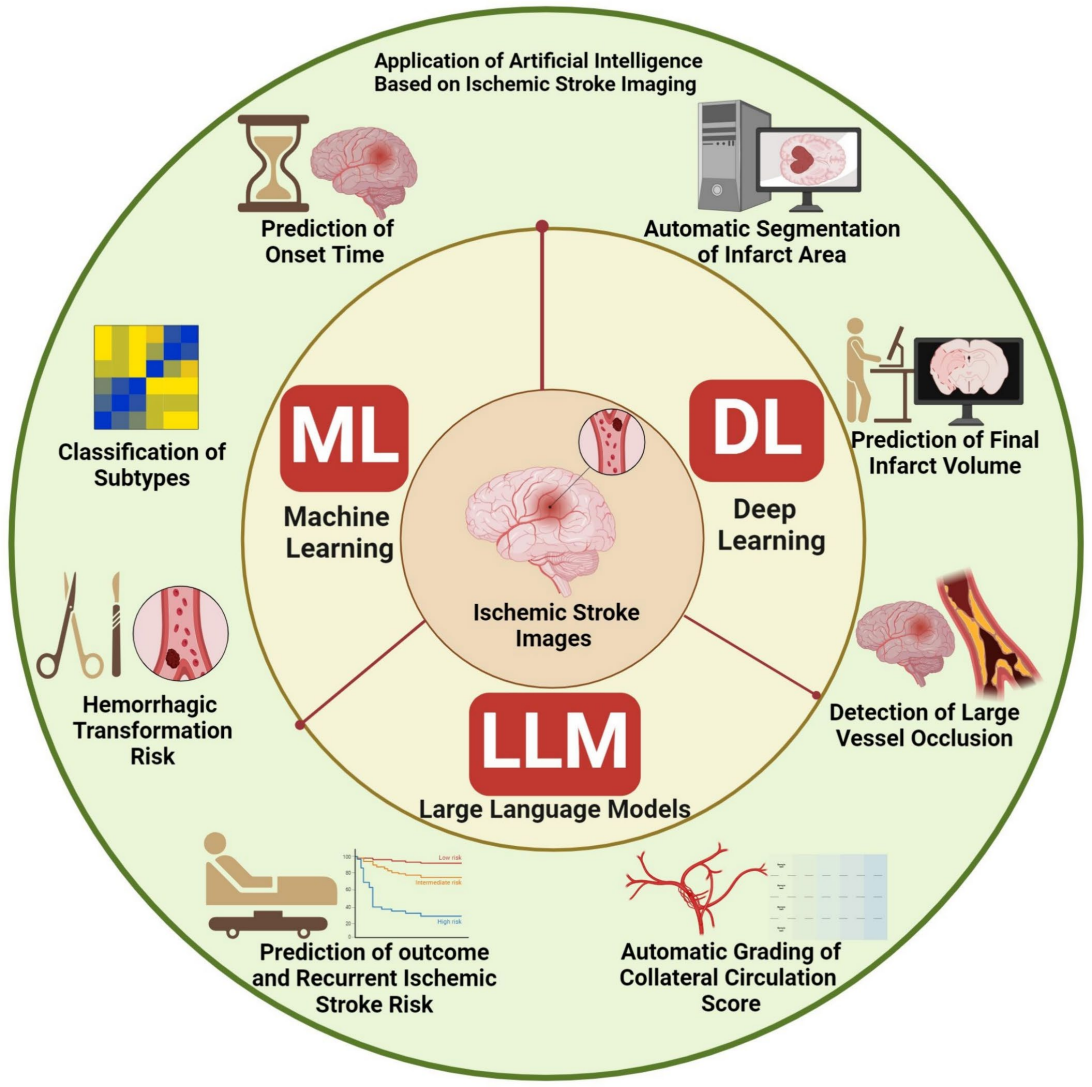
Overview of the main aspects of this review.

In this study, we aim to review the current research landscape of integrating ML and DL algorithms, and LLMs in ischemic stroke imaging. Our overarching goal is to highlight the main challenges and providing directions for future research (Figure 1).

## 2 Application of AI in ischemic stroke

### 2.1 Application of AI in the diagnosis of ischemic stroke

#### 2.1.1 Automatic segmentation of infarct area and prediction of final infarct volume

DL has gained widespread application in the segmentation of stroke images. The segmentation of stroke lesions based on neuroimaging is important in many aspects, such as quantifying the infarct volume, assessing the condition, and predicting outcomes like hemorrhagic transformation. However, in current clinical practice, manual annotation by physicians is still considered the gold standard for segmenting stroke lesions. This process is time-consuming, costly in terms of human resources, and highly dependent on the physician’s experience, which may lead to human assessment errors (8). To improve segmentation performance, DL-based methods have been proposed, with CNN-based acute ischemic stroke (AIS) infarct segmentation methods achieving excellent performance. The standard method for lesion segmentation involves thresholding CT perfusion (CTP) images. However, this approach is neither accurate nor time-consuming. Woo et al. (9) obtained DWI images of 89 patients and constructed a model using CNN. He compared this model with a traditional ML model inputting the ADC. The performance of the algorithm was evaluated using the dice coefficient in a 10-fold cross-validation, and the results revealed that the CNN algorithm for automatic segmentation of acute ischemic lesions on DWI achieved a dice coefficient of ≥0.85, outperforming traditional algorithms.

Soltanpour et al. (10) proposed a new DL-based technique called the MutiRes U-Net. Automatic segmentation of ischemic stroke lesions was achieved by enriching CTP images with contralateral and corresponding Tmax images and subsequently using them as input images for MutiRes U-Net. The study results showed a dice similarity coefficient of 0.68, indicating improved accuracy in segmentation tasks. Accurate segmentation of brain ischemia on CT images is crucial for preventing early hematoma expansion in patients with stroke (11). However, several issues remain unresolved, including images with blurred image, cavitation phenomena, and grayscale uneveness. In addition to CT and CTP images, MRI combined with DWI sequences is more sensitive for early ischemic detection (12). Juan et al. (13) utilized DWI and optimized ADC thresholds as inputs for a DL model. The results demonstrated an ICC > 0.98, indicating a high consistency between the expert manual annotations and the DL model automatic segmentation of the infarct core region. The combination of ADC thresholds and DWI achieved a higher dice similarity coefficient than DWI alone. Notably, the use of perfusion weighted images (PWI) increases the time and cost of imaging and may cause harm to patients. Utilizing only baseline DWI as input, Sanaz et al. (14) constructed a predictive model using a deep CNN and achieved a median AUC of 0.91, which implies good predictive accuracy. Thus, DL combined with DWI can predict the final infarct volume in patients with stroke, avoid overreliance on PWI to assess the final lesion volume, and lead to shorter imaging examination times and faster patient triage. These studies demonstrate the enormous potential of DL as a tool for segmenting the infarct core and predicting the final infarct volume, offering possibilities for DL to assist clinicians in quantitatively assessing lesions and choosing more effective treatment plans for patients.

With the rapid development of LLMs based on transformer architectures, powerful natural language understanding and processing capabilities have attracted widespread attention in the medical field (15). Compared with traditional supervised DL models, transformer models reduce the need for large amounts of manual annotation while also possessing greater scalability (16). Lu et al. (17) utilized a Vision Transformer (Vit) to evaluate ischemic stroke using CCD images. Through pre-trained parameters, image features can be automatically and efficiently generated without manual intervention, thereby reducing the time-consuming training process for practical clinical use.

In addition, transformers can effectively address the challenges faced by current DL models. First, traditional DL attention mechanisms typically focus only on local features and do not consider global contextual information, reducing the segmentation accuracy and precision (18). However, transformers are based on global perception through self-attention mechanisms, and along with the introduction of positional encoding and multilayer feature fusion, they can establish global dependencies between different positions and better capture the overall contextual information (19). Overall, they overcome the limitations of local feature attention structures. Second, simple down-sampling in DL leads to semantic information loss, particularly for dense prediction tasks (20). In transformers, the features at each position are obtained by the weighted summation of all positions, meaning that feature representations at each position are adaptive and can be dynamically adjusted according to the task objectives (21). This adaptability helps the model better handle tasks of different scales and complexities, reducing the loss of task-relevant semantic information caused by simple downsampling.

Therefore, the combination of transformers with traditional neural network structures provides a new approach to solving problems related to stroke imaging. By leveraging a combination of U-Net, which restores local spatial information, transformers can be used as powerful encoders for medical image segmentation tasks to address existing issues. However, the training of transformers for imaging tasks is complex and requires high-performance computers. Xu et al. (22) proposed an automatic segmentation method comprising a CNN encoder (including a Conv-IN-ReLU module and three ResConvBlocks), a transformer encoder, and a decoder. They highlighted that high accuracy will be achieved through use of CBAM enhancement to extract the importance of the CT image features. Sho et al. (23) combined U-Net neural networks with transformers to form a parallel hybrid neural network called the U-Net Transformer. The U-Net stage focused on local feature extraction and fine segmentation, whereas the transformer stage focused on capturing global dependencies and long-term correlations. The U-Net transformer integrated self-supervised learning mechanisms into the transformer network to enhance the overall segmentation and generalization capabilities. It achieved this by utilizing intermediate feature vectors from the U-Net decoder. The results showed that the U-Net transformer outperformed the state-of-the-art SPiN neural network in the MRI and CT image segmentation of lesions in patients with stroke. However, the U-Net transformer tends to incorrectly identify normal brain tissue as infarcts and ignore true lesions, indicating that simple downsampling makes the transformer structure prone to ignoring local details. To address the limitations of U-Net transformers, Wu et al. (24) proposed a novel DL architecture called the feature refinement and protection network (FRP-Net) for stroke lesion segmentation tasks. The design of the FRP-Net aims to effectively address feature refinement and information loss in lesion segmentation. The network adopts a symmetric encoder-decoder structure and integrates twin attention gates (TAG) and multidimensional attention pool (MAP) modules. FRP-Net not only accurately locates lesions through attention mechanisms but also refines lesion contours, improving the accurate identification and segmentation of lesion areas. Research findings show that its segmentation ability for stroke lesions surpassed existing state-of-the-art techniques, with dice similarity coefficients (DSC) of 60.16 and 85.72% when deployed on two ischemic stroke datasets.

In addition to basic research based on the transformer large model architecture, further study of large language models, such as GPT-4 and BERT, to analyze and interpret image processing results may show potential to provide support for clinical decision making (25).

#### 2.1.2 Detection of large vessel occlusion in ischemic stroke

AI has improved the diagnostic speed and detection rate of LVO in ischemic stroke through high-precision image analysis and data processing. Most cases of ischemic stroke are caused by acute intracranial arterial thromboembolism. Although this is seen in only 38% of ischemic stroke cases, it is responsible for 60% of all stroke-related disabilities and 90% of stroke-related deaths (26, 27). In affected patients, the likelihood of a favorable outcome decreases by 11% for every half-hour delay in effective treatment (28). Therefore, rapid and accurate detection of LVO is essential. Stavros et al. (29) utilized an automated detection software, *Viz* LVO, as an adjunct tool for stroke diagnosis and detecting LVOs based on CT angiography images. The detection rates for ICA-T, M1, and M2 occlusions were 100, 93, and 49%, respectively, which were higher than those achieved using manual clinical methods. The ability of *Viz* LVO to rapidly and accurately diagnose stroke and its high negative predictive value can reduce the number of missed diagnoses and improve diagnostic accuracy and treatment, making it a potentially valuable adjunct tool for stroke diagnosis.

Jui et al. (30) obtained the digital subtraction angiography images of 82 patients with acute ischemic stroke. They employed two neural networks, ResNet-50 pre-trained on ImageNet and ResNet-50 trained from scratch, and compared rates with two doctors identifying vessel occlusions as reference standards. The results showed that ResNet-50, trained from scratch, detected vessel occlusions more accurately, with an AUC of 0.973. The rapid and accurate diagnosis and high negative predictive value of DL algorithms contribute to the early identification and better clinical prognosis of patients (Table 1).

**Table 1:** Summary of the application of artificial intelligence in the diagnosis of ischemic stroke.

Author and year	Imaging modality	Dataset size	Methodology	Multi center	External validation	Clinical application	Evaluation metrics	Conclusion
Woo et al. (9)	MRI	429	U-Net, Dense-Net	No	No	Segmentation	U-Net: DSC = 0.85 Dense-Net: DSC =0.85	DL outperforms traditional ML models
Soltanpour et al. (10)	CTP	103	MutiRes U-Net	No	No	Segmentation	DSC = 0.68	DL improves segmentation performance.
Sanaz et al. (14)	MRI	445	DCNN	No	No	Segmentation	DSC = 0.50	DL segmentation of ischemic stroke infarct core
Xu et al. (22)	CT	379	CNN + Transformer	No	No	Segmentation	DSC = 58.66%	Achieves high-precision segmentation of CT images in ischemic stroke patients.
Sho et al. (23)	MRI, CT	239	U-Net Transformer	No	No	Segmentation	DSC = 47.2%	By combining the advantages of U-Net and Transformer, segmentation capability is enhanced.
Wu et al. (24)	MRI	240	FRPNet	No	No	Segmentation	DSC = 60.16, 85.72%	Addresses feature refinement and information loss in segmentation, improving segmentation capability.
Stavros et al. (29)	CTA	1882	AI software (*Viz* LVO)	Yes	No	Identify LVO	Detection rates = 100, 93, 49%	AI detection rates are higher than manual detection.
Jui et al. (30)	DSA	82	ResNet-50	No	No	Identify LVO	AUC = 97.3%	DL can quickly and accurately identify LVO.

DSC, Dice similarity coefficient; DCNN, deep convolutional neural network; LVO, large vessel occlusion; VGG-16, visual geometry group network with 16 layers; DCNN, Deep Convolutional Neural Network; ResNet-50, Residual Network-50; TPR, Sensitivity; AUC, Area Under Curve; FPR, Specificity; LR, logistic regression; RF, Random Forest; ML, Machine Learning; DL, Deep Learning; AI, Artificial Intelligence.

### 2.2 Application of AI in the treatment of stroke

#### 2.2.1 Identification of onset time of ischemic stroke

AI has demonstrated outstanding performance in determining the onset time of ischemic stroke, even surpassing the human DWI-FLAIR mismatch in some studies (31). Ischemic stroke requires accurate prediction of the stroke onset time (≤4.5 h) for treatment selection (32). Previous studies have shown that the development of ischemic tissue is indicated by a mismatch DWI and FLAIR. Identification of this mismatch on imaging aids in identifying potential candidates for thrombolysis. However, this method relies heavily on physician experience and may exclude many patients who qualify for treatment.

To address this issue, Hyunna et al. (32) developed three ML models, including LR, RF, and SVM, to identify the stroke onset time (≤4.5 h). Incorporating DWI and FLAIR data from 355 patients into the models showed an increased sensitivity in all three ML models when compared to physician assessments, with RF demonstrating the highest sensitivity at 75.8%, However, there was no significant difference in specificity compared to physicians, with all three models achieving a specificity of 82.6%. This highlights the potential of ML algorithms based on DWI and FLAIR features to identify the onset time of stroke and guide decision on thrombolysis. Liang et al. (33) developed ML models based on diffusion- and perfusion-weighted imaging fusion (DP fusion) to identify stroke within 4.5 h. The results revealed that DP fusion-based ML models yielded a greater net benefit than DWI- and PWI-based ML models, suggesting that in addition to selecting more advanced algorithms, integrating different imaging data could be enhance model performance.

Zhu et al. (34) employed the EfficientNet-B0 network approach for binary prediction of symptom onset time (≤4.5 h). The core methodology involved mobile inverted bottleneck convolution (MBConv) for segmentation in the DWI region of interest (ROI). To address challenges such as the delayed appearance of FLAIR infarct signals and the imbalance between lesion ROIs and other tissues, the researchers utilized a cross-modal network to provide lesion location information from DWI for FLAIR segmentation. These features were then inputted into an ML model to determine TSS. The study findings showed an accuracy of 0.805 for the model, surpassing traditional ML predictions and further validating the predictive advantage of DL in handling large datasets on nonlinear stroke lesion development.

#### 2.2.2 Classification of ischemic stroke into subtypes

AI technology based on imaging data plays a crucial role in the classification of stroke into subtypes. The combination of radiomics and ML provides a new method for accurately identifying the etiology of ischemic stroke. Accurate identification of the etiology of ischemic stroke is crucial for timely treatment to address its cause and prevent new ischemic events (35). However, identification of the etiology is often challenging and relies mainly on clinical features and data obtained through imaging techniques and other ancillary investigations. The TOAST system classifies stroke based on different etiologies and it includes five subtypes: large-artery atherosclerosis, cardioembolism, small-vessel occlusion, stroke of other determined etiologies, and stroke of undetermined etiology (36).

As an ensemble learning algorithm, RF is used for classification and regression problems, and it consists of multiple decision trees trained independently. Final prediction using RF is based on votes or the average of all trees. Zhang et al. (37) used RF combined with radiomics features to identify and classify symptomatic and asymptomatic basilar artery plaques in acute and subacute strokes. The results showed that ML model incorporating radiomic features achieved an AUC of 0.936 and an accuracy of 83.2%, demonstrating the value of ML algorithms in the classification of stroke subtypes.

Wu et al. (38) incorporated DWI data from a large database of 2,770 patients with stroke and employed DeepMedic for automation and precise lesion segmentation to distinguish different stroke subtypes. The results indicated that the performance of the ensemble model surpassed all individual CNN models, with a dice coefficient of 0.77 and a precision of 0.83. The results indicated that large artery atherosclerotic stroke had the most distinctive lesion shapes, whereas small vessel occlusion stroke had the smallest lesion areas. This suggests that DL based on extensive imaging data is valuable for stroke subtype classification and may pave way for future high-throughput studies using AI-driven tools to explore the correlations between imaging phenotypes, genetics, stroke severity, and long-term functional outcomes in large multicenter datasets.

#### 2.2.3 Automatic grading of collateral circulation score

Collateral scoring is typically based on visual assessments of neuroimaging such as CTA and CTP, which rely heavily on the radiologist’s level of expertise, resulting in significant inter-observer variability. However, DL offers a more objective computational method for clinical collateral circulation scoring, reducing observer dependency, and enhancing the consistency and accuracy of evaluations. Collateral circulation scoring is a relevant parameter for determining treatment effects and is significantly associated with postoperative hyperperfusion and recurrence (39). Kim et al. (40) developed a supervised DL model for grading the collateral circulation status in dynamic susceptibility contrast-enhanced MR perfusion images using expert manual grading scores as a reference. The results showed good consistency between DL-based collateral circulation grading and expert manual grading in both the development and validation cohorts. Current research on the use of DL for predicting collateral circulation is limited. Further prospective clinical studies are needed to verify the accuracy and reliability of DL models. Only with large-scale clinical validation can DL models become useful tools in clinical practice and provide more information and guidance for patient treatments (Table 2).

**Table 2:** Summary table of the application of artificial intelligence in the treatment of ischemic stroke.

Author and year	Imaging modality	Dataset size	Methodology	Multi center	External validation	Clinical application	Evaluation metrics	Conclusion
Hyunna et al. (32)	MRI	355	SVM, RF, LR	No	No	Identify of Onset Time	RF: TPR = 75.8% FPR = 82.6%	ML can predict the onset time, aiding doctors in choosing treatment plans.
Liang et al. (33)	MRI	433	LR, SVM	No	No	Identify of Onset Time	LR: AUC = 0.91 SVM: AUC = 0.90	Integrating different images can also improve prediction accuracy.
Zhu et al. (34)	MRI	268	EfficientNet-B0U	No	No	Identify of Onset Time	ACC = 80.5% TPR = 76.9% FPR = 84.0%	DL shows higher accuracy than ML.
Wu et al. (38)	MRI	2,770	DeepMedic	Yes	Yes	Classification of Subtypes	Precision = 0.83	AI based on imaging data can accurately classify stroke subtypes.
Zhang et al. (37)	MRI	174	RF + Radiomic	No	No	Classification of Subtypes	AUC = 0.936	Combining ML with radiomics can diagnose stroke subtypes.

TPR, Sensitivity; AUC, Area Under Curve; FPR, Specificity; SVM, Support Vector Machine; LR, logistic regression; RF, Random Forest; ML, Machine Learning; DL, Deep Learning.

### 2.3 Applications of AI in stroke outcome

#### 2.3.1 Prediction of stroke outcomes

ML has proven to be a powerful tool for predicting outcomes following ischemic stroke, and various models have been developed for this purpose. High mortality and disability rates associated with ischemic stroke impose significant economic and psychological burdens on patients. Early and accurate prognostic predictions can aid physicians in identifying high-risk patients and enable timely and personalized interventions and treatments. This can reduce unnecessary treatments and complications and facilitate effective communication among healthcare providers, patients, and their families.

Studies have shown that ML-based predictive models have higher accuracy in forecasting long-term outcomes for patients with ischemic stroke than widely used clinical scoring systems, such as the ASTRAL and SOAR scores (41). This enhanced accuracy is likely due to the complex and nonlinear relationships between the disease manifestations and clinical data. The prognosis of stroke is frequently determined by interactions of multiple factors. Unlike scoring systems and traditional statistical models that are assume a linear relationship, ML is better posed to capture the existing nonlinear relationships more effectively. By constructing multilevel data representations ranging from simple to complex, ML provides valuable insights into disease diagnosis, prognosis, and treatment. Moreover, its automated data analysis process quickly delivers more accurate results, reduces human bias, and improves prediction accuracy. Studies have also shown that in addition to patient characteristics and clinical data, details such as volume and location of the infarction are significantly associated with the outcomes of ischemic stroke. This has led to an increasing integration of medical imaging data and clinical data for outcome prediction. Zhang et al. (42) enrolled 240 patients with acute ischemic stroke who underwent standard treatment. They extracted radiomic features from the infarct region in non-contrast CT scan images and used the Kruskal–Wallis test and recursive feature elimination to select radiomic features. These features were subsequently matched with clinical characteristics and incorporated into predictive models constructed using the SVM algorithm. To enhance the model interpretability and highlight the importance of predictive features, the researchers employed the Shapley algorithm. The results indicated that the predictive model incorporating only clinical characteristics had an AUC of 0.643, which was lower than that of the model based on radiomic features alone (AUC = 0.705). The model integrating both radiomics and clinical features demonstrated the best predictive performance, with an AUC of 0.857, suggesting that ML algorithms provide high predictive accuracy for the prognosis of patients with acute ischemic stroke receiving standard treatment and can assist in early individualized care. Moreover, imaging data enhanced the predictive accuracy of ML models.

Yang et al. (7) developed a DL imaging biomarker based on MR images to predict poor outcomes 3 months following acute ischemic stroke. The research team trained a DL model using a deep neural network architecture on MR images and radiomic features to generate a DL score. The accuracy of the DL score was compared with that of five commonly used clinical risk scores (NIHSS score, SPAN, PLAN score, DSS score, and ASTRAL), and the additional benefit of the DL score to these risk scores was evaluated. The results showed no significant difference between the DL score alone and the other four risk scores; however, adding the DL score to the four risk scores improved their predictive performance.

Owing to their robust capability to capture complex relationships, transformers have been widely used for the joint processing of multimodal datasets (43). Their cross-attention mechanism allows transformer-based model to focus selectively on relevant information from different modalities and integrate it into context-aware representations (44). Furthermore, models can simultaneously consider multiple modalities and extract complementary and interrelated features, thereby enhancing its performance in multimodal tasks. Amador et al. (45) utilized an advanced spatiotemporal CNN-transformer architecture to analyze 4D CTP images. The researchers also combined 4D CTP imaging with clinical data to predict stroke lesion outcomes. The spatiotemporal CNN-transformer architecture enabled the model to effectively handle time-series data, and the introduction of the cross-attention mechanism facilitated the comprehensive modeling of spatial and temporal relationships. Finally, attention maps were generated to identify the most relevant clinical variables at the patient level, improve model interpretability, and provide clinicians with a more comprehensive understanding of the patients’ conditions.

### 2.4 Prediction of hemorrhagic transformation risk

The combination of AI and radiomics provides a reliable method for the early prediction of risk of hemorrhagic transformation, and numerous studies have explored this area. Hemorrhagic transformation is a common complication in patients with acute ischemic stroke and can occur following treatments, such as intravenous thrombolysis and mechanical thrombectomy, posing a serious threat to patient safety. Therefore, early and accurate prediction of HT risk is important. Currently, clinicians often predict risk of hemorrhagic transformation by manually assessing individual risk factors such as onset time, NIHSS score, and infarct volume on DWI (46, 47). However, given the complexity of patients’ conditions, the predictive performance of these methods is not always satisfactory (48).

Radiomics utilizes high-dimensional features from medical imaging data for analysis and prediction. It allows the extraction of numerous quantitative features that reflect the biological characteristics and pathological processes of diseases, thereby providing valuable information for diagnosis, treatment, and prognosis (49). Xie et al. (50) developed a prognostic model based on the radiomic features of the infarct area in non-enhanced CT images to predict risk of HT following acute ischemic stroke. By combining the Rad score and radiological features and employing LR, the model achieved an AUC of 0.750 in the validation cohort. Meng et al. (51) extracted radiomic features from multiparametric MRI images and constructed a predictive model using RF, which revealed an AUC of 0.871, demonstrating superior predictive performance. To predict the risk of hemorrhagic transformation after IV thrombolysis, Ren et al. (52) included 517 patients, and extracted, reduced, and selected the 12 most relevant radiomic features. In combination with five clinical variables, these features were used to build predictive models using 6 ML algorithms. The results showed that SVM exhibited a higher predictive performance, with an AUC of 0.911 in an external validation cohort. Da et al. (53) prospectively included 43 patients who underwent thrombectomy and extracted radiomic features from CT images. The researchers employed 4 different machine learning algorithms to build a predictive model to predict risk of hemorrhagic transformation within 24 h post-intervention. The naïve Bayes algorithm showed the best performance in predicting risk at 24-h (sensitivity, 1.00; specificity, 0.75; accuracy, 0.82).

Liang et al. (54) used multiparametric MRI and clinical data from 392 patients who underwent endovascular thrombectomy for ischemic stroke to construct a DL model for the early prediction of hemorrhagic transformation risk. The study initially trained the DL models using single parameters such as DWI, CBF, CBV, MTT, and TTP, and the models based on MTT and TTP performed best. The features extracted from each pre-trained single-parameter model using Inception V3 were then concatenated into one tensor. Two fully connected layers and a softmax layer were added after the concatenation layer to construct a multiparametric DL model for the classification of the presence of hemorrhagic transformation and were compared with single-parameter models. Finally, a composite model was developed and validated by combining the clinical features with multiparametric radiomics. The results showed that the ‘DMTC’ model based on DWI, MTT, TTP, and clinical features had the highest prediction accuracy, with an external validation AUC of 0.939. The proposed multiparametric DL model combining DWI, PWI, and clinical parameters demonstrated high predictive accuracy and generalizability, offering a potential tool for the pretreatment prediction of hemorrhagic transformation to assist in the perioperative management of patients with acute ischemic stroke and EVT. Ru et al. (55) constructed a weakly supervised deep learning (WSDL) model based on non-contrast CT images using multi-instance and active learning to predict hemorrhagic transformation in acute ischemic stroke. The robustness of the model was validated using threefold cross-validation and transfer learning. The researchers also analyzed and compared the WSDL model with clinical scoring systems commonly associated with non-contrast CT images (i.e., HAT and SEDAN scores) as well as with traditional DL and ML to assess the performance of the DL algorithm. The results indicated that the WSDL model exhibited the best predictive performance. Additionally, weakly supervised learning reduces the workload of manual interpretation and enables the rapid and accurate diagnosis of patients.

These studies demonstrated the superior performance of the ML and DL algorithms in predicting HT in ischemic stroke, highlighting their significant potential for clinical application. Additionally, multiple studies have shown that machine learning predictive models that combine radiomics and clinical features often exhibit superior predictive performance. These advancements indicate that machine learning, particularly when integrated with clinical insights and radiomic analysis, can significantly enhance the predictive accuracy for complications such as hemorrhagic transformation in patients with ischemic stroke. This integration not only leverages the strengths of each approach but also opens up new avenues for more personalized and effective stroke management.

### 2.5 Prediction of recurrent ischemic stroke risk

The AI-based stroke recurrence risk prediction model offers a noninvasive method for improving patients’ quality of life and reducing mortality rates. Recurrent strokes account for 25–30% of all preventable strokes, with higher disability and mortality rates than initial strokes (56). LightGBM, a machine learning algorithm based on gradient boosting decision trees, employs an efficient tree-learning algorithm to build an ensemble model quickly. Liu et al. (57) extracted radiomic features, used least absolute shrinkage and selection operator (LASSO) regression analysis to filter radiomic features, and selected 20 key radiomic features. Recursive prediction models are constructed using four ML algorithms: LR, SVM, LightGBM, and RF. For each algorithm, multiple models were built based on MRI radiomic features, clinical features, or a combination of both. The LightGBM model, which integrates radiomic and clinical features, demonstrated the best performance, with a sensitivity of 0.85, specificity of 0.805, and AUC of 0.789. By predicting the risk of recurrence in stroke patients, early detection and intervention can be implemented to maximize patient safety (Table 3).

**Table 3:** A summary table of the application of artificial intelligence in predicting prognosis in ischemic stroke.

Author and year	Imaging modality	Dataset size	Methodology	Multicenter	External validation	Clinical application	Evaluation metrics	Conclusion
Zhang et al. (42)	MRI	224	Radiomic+SVM	No	No	Prediction of outcome	AUC = 0.857	Machine learning has high predictive accuracy for outcomes in stroke patients receiving conventional treatment. Considering radiomics and clinical features enhances the model’s predictive performance.
Amador et al. (45)	4D-CTP	147	spatiotemporal CNN-Transformer	No	No	Prediction of outcome	MSE = 0.084	This work highlights the potential of the method to provide interpretable stroke treatment decision support without requiring manual annotations.
Ren et al. (52)	CT	517	XGBoost + Radiomic	Yes	Yes	Prediction of HT Risk	Internal validation: AUC = 0.950 External validation: AUC = 0.942	ML has become a tool for predicting the risk of acute hemorrhagic transformation after intravenous thrombolysis.
Xie et al. (50)	CT	118	LR + Radiomic	Yes	Yes	Prediction of HT Risk	Internal validation: AUC = 0.845 External validation: AUC = 0.750	ML supports predictive analysis of CT stroke images to achieve early prediction and intervention for hemorrhagic transformation.
Meng et al. (51)	MRI	71	RF + Radiomic	No	No	Prediction of HT Risk	AUC = 0.871	ML supports predictive analysis of hemorrhagic transformation in MRI images.
Liang et al. (54)	MRI	392	Inception V3 (CNN)	Yes	Yes	Prediction of HT Risk	Internal validation: AUC = 0.932 External validation: AUC = 0.939	The proposed multiparameter DL model has great potential for assisting the periprocedural management in the early prediction HT of the AIS patients with EVT.
Ru et al. (55)	NCCT	828	WSDL (MIL and AL)	No	No	Prediction of HT Risk	AUC = 0.799	WSDL model based on NCCT images demonstrated relatively good performance in predicting HT in AIS, reducing the cost and time associated with annotated data, and is suitable for assisting in clinical treatment decision-making.
Liu et al. (57)	MRI	612	Radiomic+ (LR, SVC, LightGBM, RF)	No	No	Prediction of Recurrent	AUC = 0.789	ML can predict the recurrence risk in stroke patients, allowing for early monitoring and intervention.

TPR, Sensitivity; AUC, Area Under Curve; FPR, Specificity; SVC, Support Vector Classification; LR, Logistic Regression; SVM, Support Vector Machine; RF, Random Forest; ACC, Accuracy; MIL, Multiple Instance Learning; AL, Active Learning. WSDL, Weakly Supervised Deep Learning; AIS, Acute Ischemic Stroke. HT, Hemorrhagic Transformation.

## 3 Current challenges and future prospects

### 3.1 Challenges in clinical translation

Ischemic stroke is an acute condition in which the decision-making speed is critical. For every hour of delay, approximately 1.9 billion neurons are lost (58). Although ML and DL models can rapidly analyze medical images, various factors in real clinical settings (such as equipment compatibility, data transmission speed, and patient cooperation) may cause a disconnect between the analysis results and the patient’s real-time condition, leading to adverse outcomes. The condition of patients with ischemic stroke can change rapidly within a short time. Therefore, models must be able to monitor, update, and learn in real time to adapt to fast-changing clinical environments.

### 3.2 Model interpretability

Ischemic stroke involves the identification, segmentation, and classification of multiple brain regions and complex changes in brain neural networks (59). Neuroradiology relies on the clinical judgment of physicians. When the ML or DL models provide a diagnostic result, doctors may require clear reasons or evidence to support the result. Therefore, there may be greater skepticism regarding black-box models (60). Although the SHAP model interpretation algorithm has been applied in multiple research fields, it is limited to explaining the contribution value of single variables in one-dimensional data, and has not yet been applied to image or multi-omics data. This limits its value in diseases such as ischemic stroke, which rely on image segmentation and recognition (61). Future algorithm development should consider the interpretability of a patient’s multimodal imaging and multi-omics indicators for clinical decision support.

### 3.3 Limitations in data volume

Machine and deep learning algorithms often require large amounts of sample data to train accurate models with robustness and generalization abilities (62). As the demand for large datasets increases, particularly with the widespread application of large language models in the medical field, the establishment of standardized large-sample databases has become more urgent. These databases provide a better foundation for multitasking and transfer learning. Models can be pre-trained on large-scale data and then transfer the learned knowledge to specific tasks, thus improving the performance in small-sample tasks.

In addition, standardized large-sample databases offer the necessary foundation for developing and validating new machine- and deep-learning algorithms. Researchers can use these databases to test algorithms, compare their effectiveness, and drive technological progress. Various factors related to ischemic stroke such as medical history, genetics, and lifestyle require substantial high-quality clinical data, particularly high-quality imaging data and comprehensive patient follow-up records. Currently, neuroimaging lacks large standardized public medical imaging databases such as The Cancer Imaging Archive (TCIA). Although there is an existing database for deep learning in ischemic stroke, ISLES has a limited sample size and lacks clinical information regarding patients. This limitation limits many studies to single centers with small sample sizes, which affects the generalizability of the models.

Furthermore, the lack of clinical information restricted the depth and quality of the research. Therefore, researchers need to establish large public databases. Despite the large number of patients with ischemic stroke worldwide and a potentially vast data pool, the lack of legal protection and regulatory mechanisms hampers effective patient privacy protection, making data sharing challenging.

Federated learning offers an innovative solution for data privacy protection and utilization problems (63). This distributed machine-learning method initializes a global model through a central server and distributes it to all participating devices. Each device uses local data to train the global model and generate local model updates. These local updates (e.g., model parameters) are then sent back to the central server without transmitting the actual data (64). The central server aggregates the updates from all the devices to obtain an updated global model, which is then redistributed to the devices (65). Throughout this process, the data remain on local devices, effectively reducing the risk of data leakage (66). As the demand for computational resources using AI technologies continues to increase and application scenarios diversify, federated learning can utilize distributed computational resources more efficiently. Furthermore, federated learning allows customized training based on the specific data of each device, thereby enhancing model adaptability and performance in specific scenarios (67). This method not only strengthens data privacy protection, but also optimizes resource utilization, providing significant technical support for a wide range of applications.

## 4 Conclusion

In conclusion, the integration of AI into ischemic stroke imaging represents a significant advancement in medical technology, offering enhanced accuracy and efficiency in diagnosing and managing stroke. These technologies show great promise in areas such as infarct segmentation, large-vessel occlusion detection, hemorrhagic transformation prediction, and stroke recurrence risk assessment. However, challenges such as the need for large and diverse datasets, interpretability of ML and DL models, and requirement for real-time processing capabilities remain obstacles to their full clinical adoption. Future progress will depend on multidisciplinary collaboration, the development of interpretable models, the establishment of comprehensive imaging databases, and continuous algorithm refinement. The potential of large language models, such as those based on the transformer architecture in stroke imaging analysis, opens up new research avenues, promising more personalized and effective stroke management strategies. Despite these challenges, the transformative potential of AI in stroke care is clear, and continued exploration and investment in these technologies are crucial to realize their full potential in improving patient outcomes.

## Author contributions

YL: Writing – original draft. ZW: Writing – original draft. YW: Writing – original draft. YZ: Writing – original draft. JW: Writing – original draft. YH: Writing – original draft. PZ: Writing – review & editing. SG: Writing – review & editing.
